# Effects of Mild Therapeutic Hypothermia on Hemodynamic Support in Cardiogenic Shock After Acute Myocardial Infarction

**DOI:** 10.7759/cureus.86407

**Published:** 2025-06-20

**Authors:** Mohamed A Abdelaal, Amany Allaithy, Taimor Mustafa, Medhat Ashmawy

**Affiliations:** 1 Cardiology, Aswan Heart Centre, Aswan, EGY; 2 Cardiology, Faculty of Medicine, Tanta University, Tanta, EGY

**Keywords:** acute myocardial infarction, cardiogenic shock, inotropes, mild therapeutic hypothermia, vasopressors

## Abstract

Background: Acute myocardial infarction (AMI) is a prevalent etiology of cardiogenic shock (CS). Microcirculatory dysfunction may continue even when hemodynamic factors improve in CS because the condition is hemodynamically diverse. Patients with CS associated with AMI have been advised to utilize vasopressors and inotropic medications. The study aims to assess the effects of mild therapeutic hypothermia (MTH) on vasopressors and inotropes in patients with CS due to AMI.

Methods: The study was carried out on a cohort of 60 individuals who had CS after AMI. CS was operationally defined as the condition characterized by a systolic blood pressure below 90 mm Hg for a duration beyond 30 minutes or the presence of inotropes required to sustain a systolic blood pressure over 90 mm Hg without any indications of hypovolemia. Vasopressor and inotrope use were guided by cardiac specialists using a predefined hemodynamic protocol. Cardiogenic shock was defined by hypotension or inotrope dependence with evidence of hypoperfusion. Mean arterial blood pressure (MAP) and lactate levels were monitored every two hours for 30 hours to ensure treatment was based on objective criteria.

Results: Norepinephrine (NE) was significantly lower at four, six, eight, 10, 12, 14, 16, 18, and 20 hours in group I than in group II (p-value < 0.001) and was insignificantly different at 0, two, 22, 24, 26, 28, and 30 hours between both groups. The dobutamine dose was significantly higher at 10 hours in group I than in group II (p-value = 0.002) and was significantly lower at 14 hours in group I than in group II (p-value = 0.036) and was insignificantly different at 0, two, four, six, eight, 12, 16, 18, 20, 22, 24, 26, 28, and 30 hours between both groups.

Conclusions: Patients treated with MTH to 33°C for 24 to 36 hours were associated with reduced NE requirements and higher MAP, along with increased dobutamine doses, compared to those without MTH. Arterial lactate rose initially with MTH but later declined. However, complications, clinical outcomes, and 30-day mortality were comparable.

## Introduction

Cardiogenic shock (CS) is a condition characterized by a severe lack of blood flow to vital organs caused by primary malfunction of the heart, as described by the European Society of Cardiology (ESC) and the American Heart Association (AHA) [1,2].

Acute myocardial infarction (AMI) is a prevalent etiology of CS, constituting a minimum of 30% of the instances. Fifty percent of CS cases manifest upon hospital admission, whereas the remaining 50% occur after hospital admission [3].

Microcirculatory dysfunction may persist even when hemodynamic factors improve in CS, as the condition is characterized by hemodynamic diversity [4]. Patients with CS associated with AMI have been advised to utilize vasopressors and inotropic medications [5,6]. Without side effects, the perfect inotrope would raise cardiac output while decreasing ventricular filling pressures and mortality. For individuals who continue to have inadequate cardiac output, inotropes are recommended, according to scientific statements. An appropriate inotrope for CS therapy is still the subject of ongoing research [7].

In CS, tissue hypoperfusion occurs as a result of decreased cardiac output even though there is enough blood volume within the blood vessels. The in-hospital mortality rate for patients with AMI is 40% due to CS, and the one-year mortality rate is 50% due to the condition, even though mechanical reperfusion techniques have generally improved and myocardial revascularization therapy has been introduced [8]. There has been an uptick in the death rate in several recent registries, which may be associated with shifts in the average age and risk factors for CS patients [9,10].

Therapeutic hypothermia (TH), sometimes referred to as targeted temperature management, is advised for unconscious patients after being resuscitated after cardiac arrest (of presumed cardiac cause) [11]. The objective is to maintain a consistent temperature within the range of 32°C to 36°C for at least 24 hours [12].

TH is a recently developed method used to maintain brain function in individuals who have been successfully resuscitated after cardiac arrest [13]. The use of 24 hours of reducing body temperature to 32 to 34°C after restoration of spontaneous circulation (ROSC) has been shown to result in a significant improvement in brain function compared to standard therapy administered after resuscitation [14]. Moreover, it is worth noting that patients who receive mild TH (MTH) experience a significant increase in their survival rate, with a reported improvement in their neurological prognosis [13]. The enhanced survival rate may be partially attributed to the maintenance of brain function. Nevertheless, a growing body of data derived from both animal species and human research indicates that MTH may potentially enhance cardiac function. Hence, it is plausible that the elevated rates of survival might be attributed to the favorable hemodynamic effects of cooling [15].

After thorough research of the literature, studies evaluating the effect of mild TH on the required doses of vasopressors and inotropes in patients with cardiogenic shock following AMI are scarce.

Therefore, this study aimed to evaluate the effect of MTH on vasopressor and inotrope requirements in patients with cardiogenic shock following acute myocardial infarction, with additional assessment of its impact on hemodynamic parameters, perfusion markers, and clinical outcomes, including duration of catecholamine support, mechanical ventilation, ICU stay, stroke incidence, and 30-day mortality.

## Materials and methods

The study was carried out on a cohort of 60 individuals who had CS after AMI. CS was operationally defined as the condition characterized by a systolic blood pressure below 90 mmHg for a duration beyond 30 minutes or the presence of inotropes required to sustain a systolic blood pressure over 90 mmHg without any indications of hypovolemia. Furthermore, individuals diagnosed with CS had indications of pulmonary congestion and poor organ perfusion, as determined by the presence of at least one of the following criteria: the patient exhibits altered cognitive function, experiences cold and moist skin, has urine output below 30 mL/h, or has arterial lactate levels over 2 mmol/L, necessitating the use of intubation and sedation.

The research was carried out between October 2020 and October 2022 after approval from the Research Ethics Committee of Tanta University, located in Tanta, Egypt (Approval number: 33716/3/20), and registration on clinicaltrials.gov (ID: NCT06947616). All patients' guardians provided written informed consent.

The exclusion criteria included those who were self-ventilated and met the existing standards for targeted temperature control, namely those who were in a state of out-of-hospital cardiac arrest with rapid onset of shock and without neurological recovery.

Randomization

Randomization was conducted using a computer-generated random sequence prepared by an independent statistician. The allocation sequence was placed into sequentially numbered, opaque, sealed envelopes by a research assistant who was not involved in patient recruitment or treatment. Each envelope was stored in a secure, locked cabinet until required. When a patient was deemed eligible for the study and provided informed consent, the next sequentially numbered envelope was retrieved and opened by an independent staff member who was not involved in patient care. The group allocation was recorded, and the empty envelope was kept as a record for reviewing purposes. The study was an open-label study due to using different techniques.

The patients were randomly classified into two equal groups. Group I received MTH to 33°C for 24 to 36 hours, and group II (control group) did not receive MTH.

Each patient underwent a comprehensive evaluation, including history taking, general and clinical examination, local cardiac examination, and echocardiography studies using a GE Vivid 7-Dimension Cardiac Ultrasound (US) (GE HealthCare, Chicago, IL, USA) phased array system. These studies were conducted to evaluate the presence of structural heart disease, the presence of resting segmental wall motion, and the assessment of the ejection fraction. The mechanical complications include ventricular septal rupture (VSR) and acute mitral regurgitation (AMR), whereas the evaluation of pulmonary congestion is conducted using chest US.

Arterial lactate, mean arterial blood pressure (MAP), and norepinephrine (NE) and dobutamine dose were assessed at 0, two, four, six, eight, 10, 12, 14, 16, 18, 20, 22, 24, 26, 28, and 30 hours.

All vasopressor and inotrope decisions in our open-label trial were made by a cardiac specialist according to the pre-specified hemodynamic protocol outlined in our methods: we defined cardiogenic shock by systolic blood pressure < 90 mmHg for > 30 minutes or need for inotropes to maintain ≥ 90 mm Hg in the absence of hypovolemia, with end-organ hypoperfusion confirmed by lactate > 2 mmol/L, urine output < 30 mL/h, altered cognition, or cold, clammy skin; under this protocol, MAP and arterial lactate were measured every two hours for 30 hours, thus ensuring that all vasoactive adjustments were driven by objective, numeric targets rather than individual clinician preference.

Coronary Angiography Data

Type of myocardial infarction (MI), extent, left main stenosis, drug-eluting stent (DES) used, thrombolysis in MI (TIMI) flow before percutaneous coronary intervention (PCI), TIMI flow after PCI, extracorporeal membrane oxygenation (ECMO), intra-aortic balloon counterpulsation usage, sepsis, pneumonia, pulmonary congestion by US, duration of mechanical ventilation and inotropic support, length of intensive care unit (ICU) stay, stroke, and mortality were assessed for both groups.

The outcome represented stroke until day 30, duration of mechanical ventilation and catecholamine support, length of ICU stay, stroke, and mortality. By comparing morbidity and mortality in both groups, morbidity (duration of mechanical ventilation, length of ICU stay, and duration of inotropic support) and mortality were insignificantly different between the two groups.

Mild therapeutic hypothermia protocol

Following PCI and transfer to the critical care unit, patients receiving MTH (group I) were provided with a commercially available cooling device (CoolGard, ZOLL Medical Corp., Chelmsford, MA, USA) to preserve their body temperature. According to the procedure, the cooling process was configured to reach the goal temperature of 33°C at the highest achievable pace. Following the attainment of the desired temperature, it was sustained for 24 hours via the use of the CoolGard system's automated temperature regulating mechanism, which operates using a central temperature sensor inside the urinary bladder. Following 24 hours, the process of rewarming was commenced at a rate of 0.25°C per hour until reaching the desired temperature of 37°C. To mitigate shivering in the MTH group, the patients underwent a treatment routine that included the administration of profound sedation and, if desired, muscle relaxation. No temperature control was implemented in patients who were randomly assigned to the control group.

Sample size calculations

The computation of the sample size was performed using G*Power 3.1.9.2 software developed by the University of Kiel in Germany. We conducted a preliminary investigation, with five instances in each group. The results revealed that the average (± standard deviation) of NE dosage at six hours was 0.15 ± 0.05 mic/kg/h in group I and 0.2 ± 0.06 mic/kg/h in group II. The sample size was determined by considering the following factors: the research had an effect size of 0.905, a confidence level of 95%, a power of 90%, and a group ratio of 1:1, and three cases were included in each group to account for potential dropout. Consequently, a total of 30 patients were recruited for each group.

Statistical analysis

IBM SPSS Statistics for Windows, Version 27 (Released 2020; IBM Corp., Armonk, NY, USA), a statistical software, was used to perform the investigation. The use of the Shapiro-Wilks test and histograms evaluated the data distribution's normality. The study used an unpaired Student's t-test to analyze the numerical data, which were presented as the mean and standard deviation (SD). Also, we used repeated measure ANOVA in comparison of two, four, six, eight, 10, 12, 14, 16, 18, 20, 22, 24, 26, 28, and 30 hours to 0 hours. The chi-square test was used to analyze the frequency and percentage of qualitative variables. A result was considered statistically significant if the two-tailed p-value was less than 0.05.

## Results

**Figure 1:**
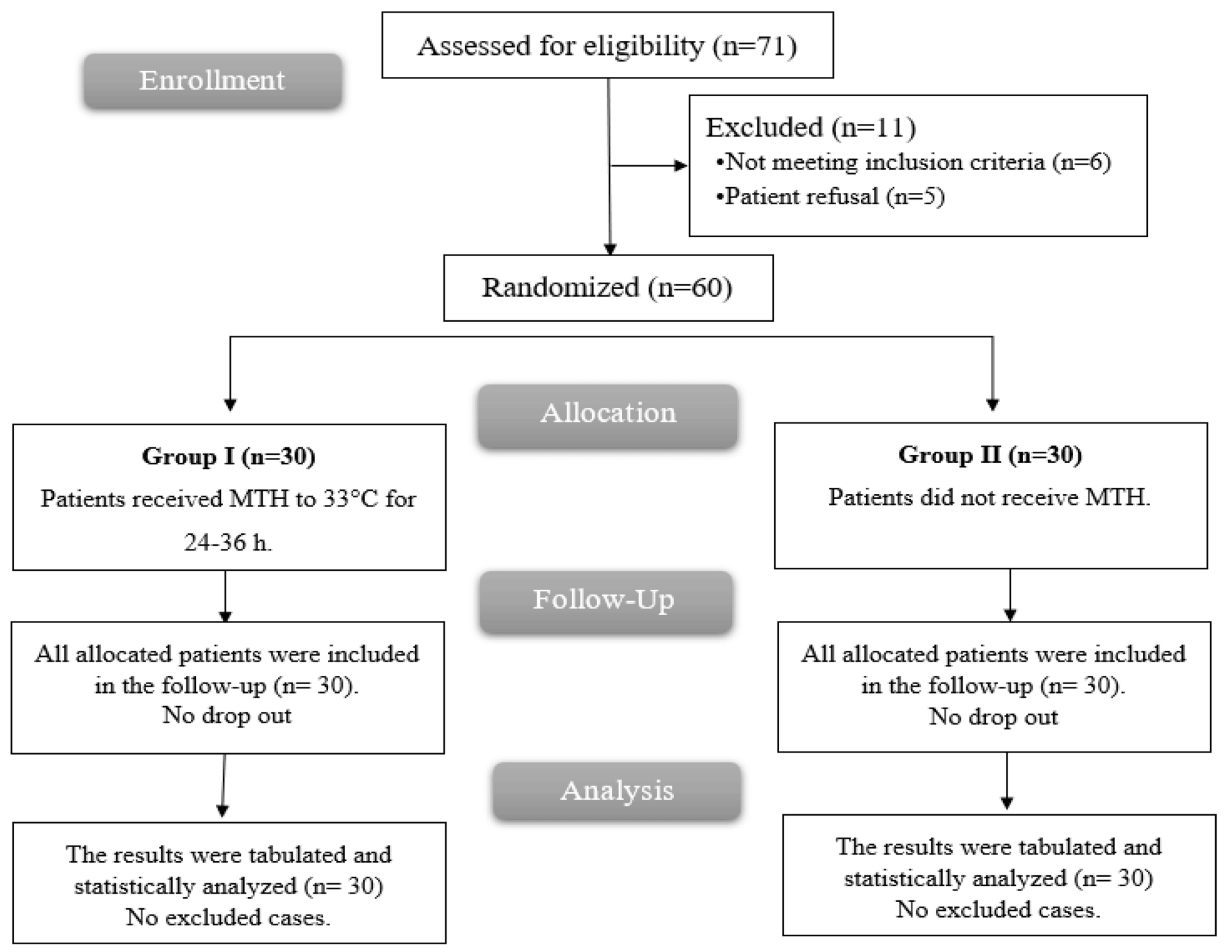
CONSORT flowchart of the enrolled patients CONSORT: Consolidated Standards of Reporting Trials; MTH: mild therapeutic hypothermia

Among the 71 patients who were considered for inclusion in the trial, five declined to take part and six did not fulfill the requirements. The remaining patients were divided into two groups of 30 each using a random allocation method. Statistical analysis was performed on all assigned subjects (Figure 1).

Patient characteristics (age, sex, and BMI) were insignificantly different between groups. Diabetes mellitus (DM), hypertension, hypercholesterolemia, smoking, prior stroke, prior myocardial infarction, and prior PCI were insignificantly different between groups (Table 1).

**Table 1:** Patient’s characteristics of studied groups Data is presented as mean ± SD or frequency (%). BMI: body mass index; HTN: hypertension; DM: diabetes mellitus; PCI: percutaneous coronary intervention; RR: relative risk; CI: confidence interval; MI: myocardial infarction

		Group I (n = 30)	Group II (n = 30)	p-value	Mean difference/RR (95% CI)
Age (years)		61.77 ± 5.05	59.87 ± 5.6	0.173	1.68 (-1.48:4.84)
Sex	Male	23 (76.7%)	22 (73.3%)	0.766	1.12 (0.79:1.58)
	Female	7 (23.3%)	8 (26.7%)		
BMI (kg/m^2^)		30.43±2.85	29.06±3.58	0.108	1.6 (-0.54:3.75)
HTN		26 (86.7%)	27 (90.0%)	0.688	0.95 (0.76:1.19)
DM		22 (73.3%)	16 (53.3%)	0.108	1.06 (0.74:1.52)
Hypercholesterolemia		18 (60%)	20 (66.67%)	0.592	0.93 (0.58:1.5)
Smoking		17 (56.7%)	13 (43.3%)	0.302	1.18 (0.66:2.11)
Prior stroke		8 (26.7%)	5 (16.7%)	0.347	1.5 (0.48:4.68)
Prior myocardial infarction		8 (26.7%)	10 (33.3%)	0.573	0.78 (0.34:1.76)
Prior PCI		6 (20.0%)	7 (23.3%)	0.754	0.86 (0.34:2.19)

Type of MI, extent, left main stenosis, DES used, TIMI flow before PCI, TIMI flow after PCI, ECMO, and intra-aortic balloon pump counterpulsation were insignificantly different between groups (Table 2).

**Table 2:** Comparison of angiographic and procedural characteristics between group I and group II Data is presented as frequency (%). MI: myocardial infarction; STEMI: ST-elevation myocardial infarction; non-STEMI: non-ST-elevation myocardial infarction; RR: relative risk; CI: confidence interval; DES: drug-eluting stent; TIMI: thrombolysis in myocardial infarction; PCI: percutaneous coronary intervention; ECMO: extracorporeal membrane oxygenation; IABP: intra-aortic balloon pump

		Group I (n = 30)	Group II (n = 30)	p-value	RR (95% CI)
Type of MI	STEMI	20 (66.7%)	18 (60.0%)	0.592	0.88 (0.58:1.34)
	Non-STEMI	10 (33.3%)	12 (40.0%)		
Extent	1 vessel	7 (23.0%)	5 (16.6%)	0.691	2 (0.56:7.12)
	2 vessel	11 (37.0%)	14 (46.7%)		
	3 vessel	12 (40.0%)	11 (36.7%)		
Left main stenosis		7 (23.3%)	4 (13.3%)	0.317	1.5 (0.48:4.68)
DES used		21 (70.0%)	22 (73.3%)	0.774	0.9 (0.68:1.2)
TIMI flow before PCI = 0		9 (30.0%)	11 (36.7%)	0.584	0.78 (0.34:1.76)
TIMI flow after PCI = 3		25 (83.3%)	26 (86. 7%)	0.718	0.95 (0.73:1.24)
ECMO		2 (6.7%)	3 (10.0%)	0.640	0.67 (0.12:3.65)
Intra-aortic balloon pump counterpulsation		13 (43.3%)	19 (63. 3%)	0.121	0.81 (0.5:1.31)

NE was significantly lower at four, six, eight, 10, 12, 14, 16, 18, and 20 hours in group I than in group II (p-value < 0.001). The total dose of NE was significantly lower in group I than in group II (p-value < 0.001).

NE was significantly lower at two, four, six, eight, 10, 12, 14, 16, 18, 20, 22, 24, 26, 28, and 30 hours compared to 0 hours (p-value < 0.001) in group I. NE was significantly lower at 12, 14, 16, 18, 20, 22, 24, 26, 28, and 30 hours compared to 0 hours in group II.

The dobutamine dose was significantly higher at 10 hours in group I than in group II (p-value = 0.002) and was significantly lower at 14 hours in group I than in group II (p-value = 0.036).

**Figure 2:**
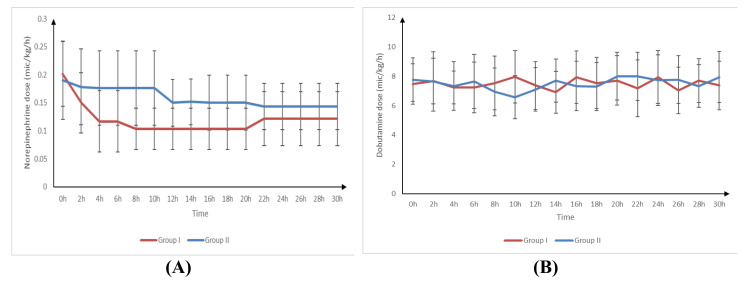
Doses of (A) norepinephrine and (B) dobutamine of studied groups

The dobutamine dose was insignificantly different between two, four, six, eight, 10, 12, 14, 16, 18, 20, 22, 24, 26, 28, and 30 hours and 0 hours in group I. The dobutamine dose was significantly lower at 10 hours than at 0 hours compared to 0 hours in group II (Table 3, Figure 2).

**Table 3:** Doses of norepinephrine (NE) and dobutamine of studied groups Data is presented as mean ± SD. *significant p-value < 0.05

	Group I (n = 30)	Group II (n = 30)	p-value	Mean difference (95% CI)
Doses of NE (mic/kg/h)				
0 hours	0.2 ± 0.06	0.19 ± 0.07	0.485	0.02 (-0.02:0.05)
2 hours	0.15 ± 0.05	0.18 ± 0.07	0.079	-0.04 (-0.07:0)
4 hours	0.12 ± 0.05	0.18 ± 0.07	<0.001*	-0.06 (-0.09:-0.03)
6 hours	0.12 ± 0.05	0.18 ± 0.07	<0.001*	-0.06 (-0.09:-0.03)
8 hours	0.1 ± 0.04	0.18 ± 0.07	<0.001*	-0.07 (-0.1:-0.04)
10 hours	0.1 ± 0.04	0.18 ± 0.07	<0.001*	-0.07 (-0.1:-0.04)
12 hours	0.1 ± 0.04	0.15 ± 0.04	<0.001*	-0.05 (-0.07:-0.02)
14 hours	0.1 ± 0.04	0.15 ± 0.04	<0.001*	-0.05 (-0.07:-0.02)
16 hours	0.1 ± 0.04	0.15 ± 0.05	<0.001*	-0.05 (-0.07:-0.02)
18 hours	0.1 ± 0.04	0.15 ± 0.05	<0.001*	-0.05 (-0.07:-0.02)
20 hours	0.1 ± 0.04	0.15 ± 0.05	<0.001*	-0.05 (-0.07:-0.02)
22 hours	0.12 ± 0.05	0.14 ± 0.04	0.067	-0.02 (-0.05:0)
24 hours	0.12 ± 0.05	0.14 ± 0.04	0.067	-0.02 (-0.05:0)
26 hours	0.12 ± 0.05	0.14 ± 0.04	0.067	-0.02 (-0.05:0)
28 hours	0.12 ± 0.05	0.14 ± 0.04	0.067	-0.02 (-0.05:0)
30 hours	0.12 ± 0.05	0.14 ± 0.04	0.067	-0.02 (-0.05:0)
Doses of dobutamine (mic/kg/h)				
0 hours	7.47 ± 1.38	7.77 ± 1.48	0.42	-0.3 (-0.98:0.66)
2 hours	7.67 ± 1.56	7.63 ± 2.01	0.943	0.04 (-0.97:1.05)
4 hours	7.23 ± 1.1	7.33 ± 1.65	0.783	-0.1 (-1.12:0.48)
6 hours	7.23 ± 1.74	7.63 ± 1.85	0.391	-0.4 (-1.13:0.89)
8 hours	7.53 ± 1.83	6.93 ± 1.62	0.184	0.6 (-0.58:1.38)
10 hours	7.97 ± 1.79	6.57 ± 1.45	0.002*	1.4 (0.19:2.13)
12 hours	7.37 ± 1.63	7.1 ± 1.47	0.508	0.27 (-0.55:1.27)
14 hours	6.9 ± 1.42	7.7 ± 1.47	0.036*	-0.8 (-1.79:-0.05)
16 hours	7.93 ± 1.78	7.33 ± 1.69	0.186	0.6 (-0.43:1.55)
18 hours	7.53 ± 1.74	7.3 ± 1.64	0.595	0.23 (-0.53:1.41)
20 hours	7.7 ± 1.68	8 ± 1.62	0.485	-0.3 (-1.06:0.9)
22 hours	7.17 ± 1.93	8 ± 1.64	0.077	-0.83 (-1.71:0.35)
24 hours	7.93 ± 1.8	7.73 ± 1.72	0.662	0.2 (-0.98:1.06)
26 hours	7.03 ± 1.59	7.77 ± 1.63	0.083	-0.74 (-1.55:0.27)
28 hours	7.7 ± 1.49	7.33 ± 1.45	0.337	0.37 (-0.24:1.44)
30 hours	7.37 ± 1.65	7.93 ± 1.74	0.201	-0.56 (-1.32:0.6)

**Figure 3:**
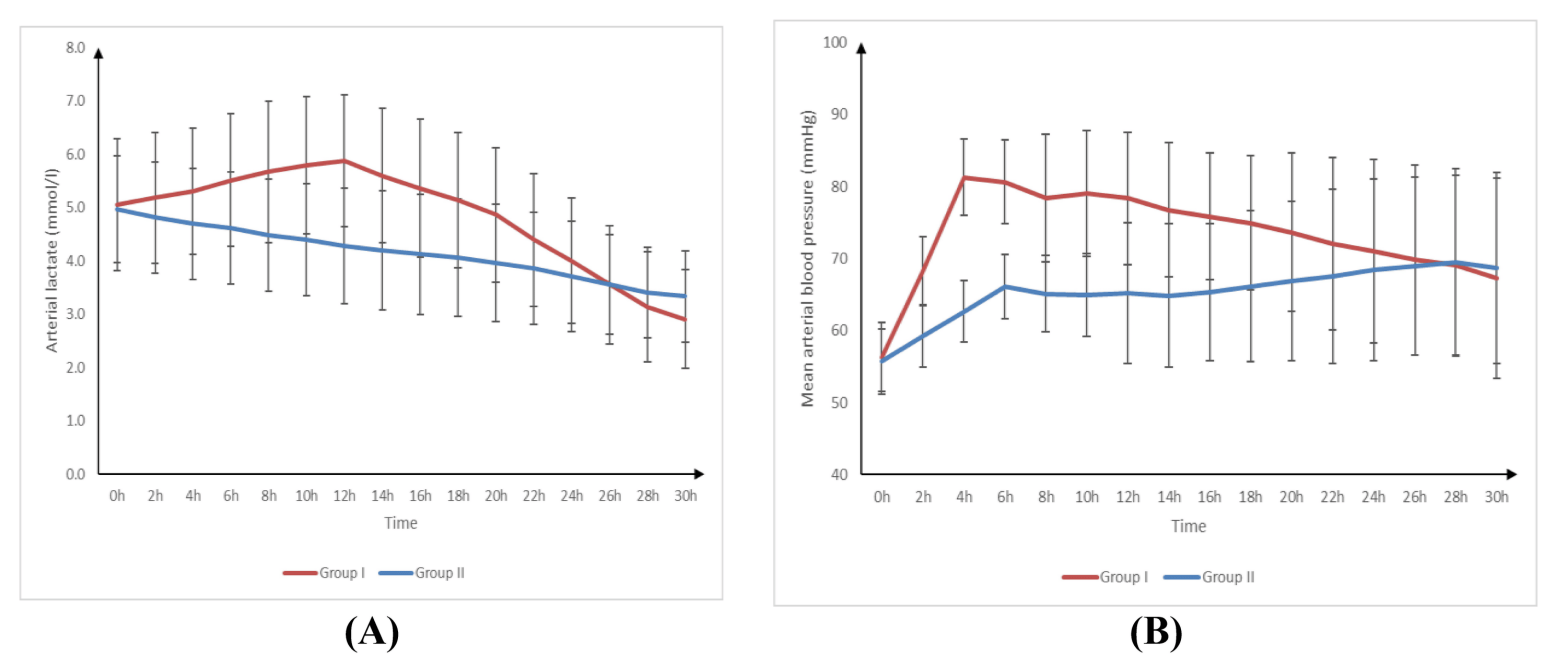
Arterial lactate (A) and mean arterial blood pressure (B) of studied groups

Arterial lactate was significantly higher at four, six, eight, 10, 12, 14, 16, 18, and 20 hours in group I than in group II (p-value < 0.05) and was insignificantly different at 0, two, 22, 24, 26, 28, and 30 hours between both groups. Arterial lactate was significantly higher at two, four, six, eight, 10, 12, and 14 hours compared to 0 hours and was significantly lower at 22, 24, 26, 28, and 30 hours compared to 0 hours (p-value < 0.05) and was insignificantly different between 18 and 20 hours and 0 hours in group I. Arterial lactate was significantly lower at four, six, eight, 10, 12, 14, 16, 18, 20, 22, 24, 26, 28, and 30 hours compared to 0 hours (p-value < 0.001) and was insignificantly different between two hours and 0 hours in group II. MAP was significantly higher at two, four, six, eight, 10, 12, 14, 16, 18, and 20 hours in group I than in group II (p-value < 0.05) and was insignificantly different at 0, 22, 24, 26, 28, and 30 hours between both groups. MAP was significantly higher at two, four, six, eight, 10, 12, 14, 16, 18, 20, 22, 24, 26, 28, and 30 hours compared to 0 hours (p-value < 0.001) in group I and group II (Table 4, Figure 3).

**Table 4:** Arterial lactate and mean arterial blood pressure (MAP) of studied groups Data is presented as mean ± SD. *significant p-value < 0.05

	Group I (n = 30)	Group II (n = 30)	p-value	Mean difference (95% CI)
Arterial lactate (mmol/l)				
0 hours	5.06 ± 1.23	4.96 ± 1	0.749	0.1 (-0.56:0.71)
2 hours	5.18 ± 1.22	4.81 ± 1.04	0.216	0.37 (-0.34:0.93)
4 hours	5.31 ± 1.19	4.7 ± 1.05	0.038	0.61 (0.06:1.32)
6 hours	5.51 ± 1.24	4.62 ± 1.05	0.004*	0.89 (0.26:1.56)
8 hours	5.67 ± 1.32	4.48 ± 1.05	<0.001*	1.19 (0.53:1.88)
10 hours	5.79 ± 1.28	4.4 ± 1.05	<0.001*	1.39 (0.74:2.08)
12 hours	5.87 ± 1.24	4.28 ± 1.08	<0.001*	1.59 (0.97:2.27)
14 hours	5.6 ± 1.27	4.2 ± 1.12	<0.001*	1.4 (0.78:2.14)
16 hours	5.36 ± 1.3	4.12 ± 1.13	<0.001*	1.24 (0.62:2.01)
18 hours	5.13 ± 1.27	4.06 ± 1.1	0.001*	1.07 (0.45:1.79)
20 hours	4.86 ± 1.26	3.96 ± 1.1	0.005*	0.9 (0.27:1.63)
22 hours	4.39 ± 1.24	3.86 ± 1.05	0.081	0.53 (-0.08:1.24)
24 hours	4 ± 1.18	3.71 ± 1.03	0.31	0.29 (-0.3:0.97)
26 hours	3.55 ± 1.11	3.56 ± 0.93	0.98	-0.01 (-0.56:0.62)
28 hours	3.14 ± 1.03	3.41 ± 0.84	0.271	-0.27 (-0.78:0.31)
30 hours	2.91 ± 0.92	3.33 ± 0.85	0.068	-0.42 (-0.7:0.31)
MAP (mmHg)				
0 hours	56.33 ± 4.78	55.73 ± 4.54	0.620	0.6 (-2.09:3.29)
2 hours	68.33 ± 4.71	59.2 ± 4.28	<0.001*	9.13 (7.01:12.03)
4 hours	81.27 ± 5.3	62.63 ± 4.27	<0.001*	18.64 (16.4:21.6)
6 hours	80.63 ± 5.81	66.13 ± 4.5	<0.001*	14.5 (11.86:17.58)
8 hours	78.43 ± 8.87	65.13 ± 5.29	<0.001*	13.3 (9.88:17.96)
10 hours	79.03 ± 8.73	64.9 ± 5.74	<0.001*	14.13 (10.76:18.76)
12 hours	78.33 ± 9.17	65.23 ± 9.73	<0.001*	13.1 (7.92:18.24)
14 hours	76.77 ± 9.28	64.87 ± 9.92	<0.001*	11.9 (6.17:16.87)
16 hours	75.87 ± 8.84	65.33 ± 9.52	<0.001*	10.54 (4.93:15.15)
18 hours	74.93 ± 9.33	66.13 ± 10.48	0.001*	8.8 (2.82:13.82)
20 hours	73.67 ± 11.03	66.87 ± 11.05	0.02*	6.8 (0.43:12.45)
22 hours	72.03 ± 11.96	67.57 ± 12.09	0.156	4.46 (-4.1:8.82)
24 hours	71 ± 12.71	68.43 ± 12.58	0.435	2.57 (-4.82:9.22)
26 hours	69.8 ± 13.14	68.97 ± 12.3	0.801	0.83 (-6.85:7.25)
28 hours	69.07 ± 12.51	69.5 ± 12.97	0.896	-0.43 (-8.65:5.69)
30 hours	67.27 ± 13.92	68.67 ± 13.28	0.692	-1.4 (-9.89:5.17)

Sepsis, pneumonia, pulmonary congestion in the US, duration of mechanical ventilation, duration of inotropic support, length of ICU stays, stroke until day 30, and mortality were insignificantly different between groups (Table 5).

**Table 5:** Outcomes of the studied groups Data is presented as mean ± SD or frequency (%). RR: relative risk; CI: confidence interval; US: ultrasound; ICU: intensive care unit

	Group I (n = 30)	Group II (n = 30)	p-value	Mean difference/RR (95% CI)
Sepsis	3 (10.0%)	1 (3.3%)	0.301	3 (0.33:26.92)
Pneumonia	9 (30.0%)	11 (36.7%)	0.584	0.82 (0.41:1.62)
Pulmonary congestion in US (3B lines or more)	23 (76.7%)	20 (66.7%)	0.390	1.15 (0.92:1.44)
Duration of mechanical ventilation (days)	4.8 ± 2.33	5.5 ± 2.27	0.243	-0.7 (-1.89:0.49)
Duration of inotropic support (days)	3.87 ± 2.39	4.93 ± 2.26	0.081	-1.06 (-2.26:-0.14)
Length of ICU stay (days)	6.9 ± 3.17	7.1 ± 2.67	0.792	-0.2 (-1.71:1.31)
Stroke	0 (0.0%)	3 (10.0%)	0.076	-
Mortality	14 (46.7%)	20 (66.7%)	0.118	0.7 (0.47:1.04)

## Discussion

This study demonstrated that NE requirements were significantly lower at multiple time points (four, six, eight, 10, 12, 14, 16, 18, and 20 hours) in group I than in group II.

Dobutamine dosing also showed a dynamic pattern; it was significantly higher at 10 hours in group I but then significantly lower at 14 hours. These findings suggest that MTH may initially influence inotropic support but contributes to improved hemodynamic stability over time.

The physiological rationale for these findings is supported by prior studies highlighting the beneficial effects of TH in the context of CS. Moderate hypothermia has been shown to enhance cardiac contractility in isolated cardiac muscle preparations and improve stroke volume and cardiac output in vivo [16]. Moreover, MTH provides cardioprotective effects by preserving myocardial function, improving microcirculation, and attenuating systemic inflammatory responses, which can exacerbate vasodilation and hypotension in CS [17].

Our literature employed a cooling duration of 24 to 36 hours, consistent with prior studies. Fuernau et al. [18] used a protocol of 24 hours at 33°C in patients with CS complicating AMI. Also, a review article by Polderman [19] discussed various aspects of TH, including duration, mentioning that 24 to 48 hours is commonly used in clinical practice.

The duration of 24 to 36 hours for MTH was selected based on previous research, although the optimal duration is still debated. Conversely, Nielsen et al. [20] stated that MTH applied for 24 hours can improve neurological outcomes in patients’ post-cardiac arrest. However, extending this cooling period slightly to 36 hours could reveal additional benefits or determine optimal duration without significantly increasing risks, as suggested by prior safety data from studies such as the Hypothermia After Cardiac Arrest (HACA) trial. Bernard et al. [21] also exhibited that a 24- to 36-hour cooling window is a practical consideration, aligning with the critical care settings' operational capabilities and ensuring that TTM is manageable and safe for implementation.

In our study, MAP at two, four, six, eight, 10, 12, 14, 16, 18, and 20 hours was significantly increased in group I compared to group II. As opposed to our results, Young et al. [22] included 188 consecutive patients who underwent ROSC after a cardiac arrest and received TH. For the first 24 hours after ROSC, all patients were actively rewarmed at a rate of 0.25°C per hour after being cooled externally using an active surface-cooling device to keep their core body temperature between 32°C and 34°C.

Our institution's standardized TH procedure suggests starting with NE for treating hypotension and aiming for a MAP goal of >65 mmHg. Before and during TH, participants' MAPs were comparable (80.3 vs. 83.7 mmHg; p = 0.11), according to the research. The study's design and the higher sample size may be associated with this discrepancy. While we found a difference in MAP between the groups treated with MTH and the control group, Fuernau et al. [16] found no such difference. The discrepancy in findings may be attributable to differences in the timing of measurements, as their study assessed outcomes over a 24-hour period, whereas our study extended the observation to 30 hours. Additionally, variations in baseline characteristics, including a higher prevalence of smoking and increased mortality rates in their study population compared to ours, may have further contributed to the observed differences.

In our study, NE at two, four, six, eight, 10, 12, 14, 16, 18, and 20 hours was significantly decreased in group I compared to group II. Also, Zobel et al. [15] documented that the usage of catecholamines was dramatically reduced in patients with AMI who were treated for hypothermia as a result of the effects of TH and pharmacological inotropes. Without the implementation of maternal treatment, a significant majority of patients (80%) required increased dosages of norepinephrine. In the control group, the infusion rate of NE exhibited a significant reduction upon induction of hypothermia.

In the current investigation, it was observed that the dobutamine dosage at 10 hours was considerably higher in group I compared to group II. Conversely, after 14 hours, the dobutamine dose was significantly lower in group I compared to group II.

MTH, typically maintained at 32 to 34°C, is known to reduce systemic metabolism, oxygen consumption, and inflammatory responses [23,24], which are beneficial in post-cardiac arrest care. However, in the context of cardiogenic shock, hypothermia introduces hemodynamic challenges that often necessitate increased inotropic support [25]. Several studies and reviews have demonstrated that MTH leads to a decrease in heart rate (bradycardia) [26] and myocardial contractility [27] due to its direct negative chronotropic and inotropic effects on the myocardium [26].

Comparable to our results, Fuernau et al. [18] highlighted that dobutamine had a faster decline in the MTH group than the control group. Also, Jacobshagen et al. [28] demonstrated that the dobutamine application rates did not change significantly before and after hypothermia (p = 0.23). A retrospective analysis of human data comparing 20 consecutive patients with CS who underwent MTH following resuscitation with a control group that was matched based on previous propensity scores revealed an elevation in MAP and reduced cumulative doses of vasopressors. However, no significant changes occurred in inotropes [3]. Furthermore, the MTH group had superior lactate clearance. On the other hand, a notably decelerated decrease in lactate levels was found in the MTH group.

Our results demonstrated that DM increased significantly in group I compared to group II. In line with our findings, Annborn et al. [29] revealed that the DM was not significantly different between the two studied groups.

Research indicates that the pharmacodynamic effects of adrenaline are not independent of temperature changes. In normothermic conditions, adrenaline enhances cardiac contraction and heart rate and can either decrease (at low doses) or increase (at high doses) systemic vascular resistance (SVR). However, during hypothermia, the response to adrenaline can be significantly altered. For instance, doses of adrenaline that induce vasodilation in normothermic conditions can increase SVR during hypothermia. This altered response is also reflected in the inotropic effect of adrenaline, which is reduced at lower temperatures [30].

Furthermore, studies have shown that during hypothermia, the positive cardiac effects of low-dose adrenaline may be observed, whereas high-dose adrenaline does not yield beneficial effects and can potentially be harmful. Hypothermia has a substantial influence on the inotropic effects on the heart that are mediated by the β1-receptor pathway, according to this. Possible causes of adrenaline's adverse effects at low temperatures include hypothermia-induced calcium overload, altered α-receptor or β2-receptor agonism, and enhanced or decreased α-conduction. These findings indicate a narrowed therapeutic window for adrenaline during hypothermia, suggesting caution in its use under these conditions. The altered pharmacodynamic effects of adrenaline during hypothermia highlight the need for careful consideration of dosage and the overall clinical condition when administering adrenaline in a hypothermic state [30].

In our study, stroke until day 30, duration of mechanical ventilation, length of ICU stay, duration of catecholamine support, and mortality were insignificantly different between both groups. Conforming to our results, Annborn et al. [29] revealed that there was no significant difference in ICU and 30-day mortality between targeted temperature control at 33°C or 36°C.

In contrast to our findings, Pavlov et al. [31] observed that TH is related to improved survival rates, indicating that TH is an essential therapy choice for survivors of comatose out-of-hospital cardiac arrest (OHCA). The variation in our results may be attributed to the varying conditions of different individuals.

In the present study, arterial lactate at four, six, eight, 10, 12, 14, 16, 18, and 20 hours was significantly increased in group I compared to group II (p < 0.05), and it was insignificantly different between both groups at 0, two, 22, 24, 26, 28, and 30 hours. In contrast, Fuernau et al.'s [18] results declared that arterial lactate decreased over time in both groups, with a slower and flatter decrease in the MTH group. The large sample size may clarify this difference.

Limitations

Our study was conducted in a single center with a small sample size. The small preliminary study and resulting sample size are limitations that may impact the study's ability to detect smaller but meaningful differences between groups and lack of protocolized hemodynamic management. A larger, multi-center study would likely be needed to evaluate the effects of MTH on this patient population more definitively to standardize protocol for hemodynamic management, especially vasopressor and inotrope titration, and their potential impact on the primary outcomes.

## Conclusions

In patients with CS following acute myocardial infarction, mild therapeutic hypothermia maintained at 33°C for 24 to 36 hours was associated with reduced norepinephrine requirements, improved mean arterial pressure, and a dynamic but ultimately decreased need for dobutamine support. Although clinical outcomes such as mechanical ventilation duration, ICU stay, and 30-day mortality were not significantly different, the hemodynamic improvements suggest that MTH may offer valuable support in the early stabilization phase of cardiogenic shock. These findings highlight MTH as a promising adjunctive strategy in critical care settings, potentially reducing vasopressor dependence and improving cardiovascular performance during the acute phase of shock management.

## References

[1] van Diepen S, Katz JN, Albert NM (2017). Contemporary management of cardiogenic shock: a scientific statement from the American Heart Association. Circulation.

[2] Ibanez B, James S, Agewall S (2018). 2017 ESC Guidelines for the management of acute myocardial infarction in patients presenting with ST-segment elevation: the task force for the management of acute myocardial infarction in patients presenting with ST-segment elevation of the European Society of Cardiology (ESC). Eur Heart J.

[3] Senman B, Jentzer JC, Barnett CF (2024). Need for a cardiogenic shock team collaborative - promoting a team‐based model of care to improve outcomes and identify best practices. J Am Heart Assoc.

[4] Crea F, Montone RA, Rinaldi R (2022). Pathophysiology of coronary microvascular dysfunction. Circ J.

[5] Uhlig K, Efremov L, Tongers J, Frantz S, Mikolajczyk R, Sedding D, Schumann J (2020). Inotropic agents and vasodilator strategies for the treatment of cardiogenic shock or low cardiac output syndrome. Cochrane Database Syst Rev.

[6] Olarte N, Rivera NT, Grazette L (2022). Evolving presentation of cardiogenic shock: a review of the medical literature and current practices. Cardiol Ther.

[7] Wang MT, Hung CC, Huang WC Medications in cardiogenic shock. Primary Angioplasty.

[8] Thiele H, Ohman EM, de Waha-Thiele S, Zeymer U, Desch S (2019). Management of cardiogenic shock complicating myocardial infarction: an update 2019. Eur Heart J.

[9] De Luca L, Savonitto S (2020). Composite trends of cardiogenic shock complicating acute myocardial infarction. Eur J Heart Fail.

[10] De Luca L, Mistrulli R, Scirpa R, Thiele H, De Luca G (2023). Contemporary management of cardiogenic shock complicating acute myocardial infarction. J Clin Med.

[11] Oprita B, Olaru I, Botezatu L, Diaconu AE, Oprita R (2025). Management of severe hypothermia: challenges and advanced strategies. J Clin Med.

[12] Hamzaoui O, Boissier F (2023). Hemodynamic monitoring in cardiogenic shock. J Intensive Med.

[13] Sandroni C, Natalini D, Nolan JP (2022). Temperature control after cardiac arrest. Crit Care.

[14] Li P, Sun Z, Tian T, Yu D, Tian H, Gong P (2023). Recent developments and controversies in therapeutic hypothermia after cardiopulmonary resuscitation. Am J Emerg Med.

[15] Zobel C, Adler C, Kranz A (2012). Mild therapeutic hypothermia in cardiogenic shock syndrome. Crit Care Med.

[16] Jung KT, Bapat A, Kim YK, Hucker WJ, Lee K (2022). Therapeutic hypothermia for acute myocardial infarction: a narrative review of evidence from animal and clinical studies. Korean J Anesthesiol.

[17] Kohlhauer M, Berdeaux A, Ghaleh B, Tissier R (2016). Therapeutic hypothermia to protect the heart against acute myocardial infarction. Arch Cardiovasc Dis.

[18] Fuernau G, Beck J, Desch S (2019). Mild hypothermia in cardiogenic shock complicating myocardial infarction: randomized SHOCK-COOL trial. Circulation.

[19] Polderman KH (2015). How to stay cool in the intensive care unit?: endovascular versus surface cooling. Circulation.

[20] Nielsen N, Wetterslev J, Cronberg T (2013). Targeted temperature management at 33°C versus 36°C after cardiac arrest. N Engl J Med.

[21] Bernard SA, Gray TW, Buist MD, Jones BM, Silvester W, Gutteridge G, Smith K (2002). Treatment of comatose survivors of out-of-hospital cardiac arrest with induced hypothermia. N Engl J Med.

[22] Young MN, Hollenbeck RD, Pollock JS, Giuseffi JL, Wang L, Harrell FE, McPherson JA (2015). Higher achieved mean arterial pressure during therapeutic hypothermia is not associated with neurologically intact survival following cardiac arrest. Resuscitation.

[23] Liu H, Zhou M (2023). The utility of therapeutic hypothermia on cerebral autoregulation. J Intensive Med.

[24] You JS, Kim JY, Yenari MA (2022). Therapeutic hypothermia for stroke: unique challenges at the bedside. Front Neurol.

[25] Preuß S, Multmeier J, Stenzel W (2024). Survival, but not the severity of hypoxic-ischemic encephalopathy, is associated with higher mean arterial blood pressure after cardiac arrest: a retrospective cohort study. Front Cardiovasc Med.

[26] Giraud R, Siegenthaler N, Bendjelid K (2013). Cardiac index during therapeutic hypothermia: which target value is optimal?. Crit Care.

[27] Kelly FE, Nolan JP (2010). The effects of mild induced hypothermia on the myocardium: a systematic review. Anaesthesia.

[28] Jacobshagen C, Pelster T, Pax A (2010). Effects of mild hypothermia on hemodynamics in cardiac arrest survivors and isolated failing human myocardium. Clin Res Cardiol.

[29] Annborn M, Bro-Jeppesen J, Nielsen N (2014). The association of targeted temperature management at 33 and 36 °C with outcome in patients with moderate shock on admission after out-of-hospital cardiac arrest: a post hoc analysis of the Target Temperature Management trial. Intensive Care Med.

[30] Dietrichs ES, Sager G, Tveita T (2016). Altered pharmacological effects of adrenergic agonists during hypothermia. Scand J Trauma Resusc Emerg Med.

[31] Pavlov M, Babić Z, Đuzel A, Crljenko K, Nedić M, Delić Brkljačić D (2020). The influence of therapeutic hypothermia on the outcomes of cardiac arrest survivors: a retrospective cohort study. Croat Med J.

